# CSF metabolites associated with biomarkers of Alzheimer’s disease pathology

**DOI:** 10.3389/fnagi.2023.1214932

**Published:** 2023-08-30

**Authors:** Ruocheng Dong, Qiongshi Lu, Hyunseung Kang, Ivonne Suridjan, Gwendlyn Kollmorgen, Norbert Wild, Yuetiva Deming, Carol A. Van Hulle, Rozalyn M. Anderson, Henrik Zetterberg, Kaj Blennow, Cynthia M. Carlsson, Sanjay Asthana, Sterling C. Johnson, Corinne D. Engelman

**Affiliations:** ^1^Department of Population Health Sciences, School of Medicine and Public Health, University of Wisconsin-Madison, Madison, WI, United States; ^2^Department of Biostatistics and Medical Informatics, School of Medicine and Public Health, University of Wisconsin-Madison, Madison, WI, United States; ^3^Roche Diagnostics International Ltd., Rotkreuz, Switzerland; ^4^Roche Diagnostics GmbH, Penzberg, Germany; ^5^Department of Medicine, School of Medicine and Public Health, University of Wisconsin-Madison, Madison, WI, United States; ^6^Wisconsin Alzheimer’s Disease Research Center, School of Medicine and Public Health, University of Wisconsin-Madison, Madison, WI, United States; ^7^Geriatrics Research Education and Clinical Center, Middleton VA Hospital, Madison, WI, United States; ^8^Department of Psychiatry and Neurochemistry, Institute of Neuroscience and Physiology, The Sahlgrenska Academy at University of Gothenburg, Mölndal, Sweden; ^9^Clinical Neurochemistry Laboratory, Sahlgrenska University Hospital, Mölndal, Sweden; ^10^UK Dementia Research Institute at UCL, London, United Kingdom; ^11^Department of Neurodegenerative Disease, UCL Institute of Neurology, London, United Kingdom; ^12^Hong Kong Center for Neurodegenerative Diseases, Hong Kong, Hong Kong SAR, China; ^13^Wisconsin Alzheimer’s Institute, School of Medicine and Public Health, University of Wisconsin-Madison, Madison, WI, United States

**Keywords:** Alzheimer’s disease, metabolomics, CSF NeuroToolKit biomarkers, metabolome-wide association, Mendelian randomization

## Abstract

**Introduction:**

Metabolomics technology facilitates studying associations between small molecules and disease processes. Correlating metabolites in cerebrospinal fluid (CSF) with Alzheimer’s disease (AD) CSF biomarkers may elucidate additional changes that are associated with early AD pathology and enhance our knowledge of the disease.

**Methods:**

The relative abundance of untargeted metabolites was assessed in 161 individuals from the Wisconsin Registry for Alzheimer’s Prevention. A metabolome-wide association study (MWAS) was conducted between 269 CSF metabolites and protein biomarkers reflecting brain amyloidosis, tau pathology, neuronal and synaptic degeneration, and astrocyte or microglial activation and neuroinflammation. Linear mixed-effects regression analyses were performed with random intercepts for sample relatedness and repeated measurements and fixed effects for age, sex, and years of education. The metabolome-wide significance was determined by a false discovery rate threshold of 0.05. The significant metabolites were replicated in 154 independent individuals from then Wisconsin Alzheimer’s Disease Research Center. Mendelian randomization was performed using genome-wide significant single nucleotide polymorphisms from a CSF metabolites genome-wide association study.

**Results:**

Metabolome-wide association study results showed several significantly associated metabolites for all the biomarkers except Aβ42/40 and IL-6. Genetic variants associated with metabolites and Mendelian randomization analysis provided evidence for a causal association of metabolites for soluble triggering receptor expressed on myeloid cells 2 (sTREM2), amyloid β (Aβ40), α-synuclein, total tau, phosphorylated tau, and neurogranin, for example, palmitoyl sphingomyelin (d18:1/16:0) for sTREM2, and erythritol for Aβ40 and α-synuclein.

**Discussion:**

This study provides evidence that CSF metabolites are associated with AD-related pathology, and many of these associations may be causal.

## Introduction

The neuropathological changes of Alzheimer’s disease (AD) consist of extracellular amyloid-β (Aβ) plaques and intracellular neurofibrillary tangles of hyperphosphorylated tau proteins in the brain (Anoop et al., 2010). Well-established core biomarkers that reflect AD pathology and show promising performance in evaluating AD risk and diagnosing AD are the 42 amino acid form Aβ (Aβ42), the ratio of Aβ42/40, phosphorylated tau (P-tau), and total tau (T-tau) in the cerebrospinal fluid (CSF) (Blennow et al., 2012). However, it has been suggested that other pathophysiology such as neuroinflammation through glial activation and neuronal and synaptic degeneration also contribute to symptomatic AD, and CSF biomarkers of these may provide valuable information about disease progression (Blennow et al., 2012). Thus, the NeuroToolKit (NTK), a panel of automated CSF immunoassays, was introduced to complement the established core AD biomarkers (Hulle et al., 2021). The NTK panel includes S100 calcium-binding protein B (S100b), chitinase-3-like protein 1 (YKL-40), and glial fibrillary acidic protein (GFAP) as markers of astrocyte activation; soluble triggering receptor expressed on myeloid cells 2 (sTREM2) and interleukin-6 (IL-6) as markers of microglial activation and inflammation; and neurofilament light (NfL), neurogranin, and α-synuclein as markers of axonal injury and synaptic dysfunction (ALZFORUM, 2019).

Untargeted metabolomics technology is a promising approach that can simultaneously identify and quantify a large number of small molecules (<1,500 Da, e.g., lipids) in a biological sample (Hasin et al., 2017). Previous research has evaluated the potential application of metabolites as biomarkers for AD and shown that metabolomic changes in the human brain and CSF were associated with AD status and AD pathological alterations (Koal et al., 2015; Jacobs et al., 2019; van der Velpen et al., 2019; Morrow et al., 2021; Dong et al., 2022; Liang et al., 2022). For example, Koal et al. (2015) identified eight metabolites that were significantly increased in the CSF samples with AD-like pathology including an acylcarnitine (C3), two sphingomyelins [SM (d18:1/18:0) and SM (d18:1/18:1)], and five glycerophospholipids (PC aa C32:0, PC aa C34:1, PC aa C36:1, PC aa C38:4, and PC aa C38:6). Recent studies focused on 12 CSF sphingomyelin metabolites (SM) (Morrow et al., 2021) or derived principal components from 308 CSF metabolites (Dong et al., 2022) also suggested evidence of an association between CSF metabolites and AD biomarkers, e.g., SM (d18:1/14:0, d16:1/16:0) and p-tau, NFL, and α -synuclein. However, no studies have examined associations between the full untargeted CSF metabolome and a broad panel of biomarkers such as the NTK panel. Thus, our study aims to link CSF metabolites with established and developing AD biomarkers with the goals of (1) identifying CSF metabolites that are individually associated with the CSF NTK biomarkers and (2) conducting Mendelian randomization (MR) to determine if the CSF metabolites significantly associated with NTK biomarkers are likely to be in the causal pathway instead of simply changing with, or as a result of, AD biomarker changes.

## Materials and methods

### Participants

The Wisconsin Registry for Alzheimer’s Prevention (WRAP) began recruitment in 2001 as a prospective cohort study, with initial follow-up 4 years after baseline and subsequent ongoing follow-up every 2 years. WRAP is comprised of initially cognitively-unimpaired, asymptomatic, middle-aged (between 40 and 65) adults enriched for parental history of clinical AD (Johnson et al., 2018). At each visit, the participants undergo comprehensive medical and cognitive evaluations. Additional details of the study design and methods of WRAP have been described previously (Johnson et al., 2018). From the WRAP cohort, we identified 161 self-reported non-Hispanic white individuals with both longitudinal CSF biomarker and metabolomic data available from 2010 to 2017. The sample size for other racial/ethnic groups was too small (*n* < 10) to include in the analyses.

The Wisconsin Alzheimer’s Disease Research Center’s (ADRC) clinical core cohorts started in 2009 and are comprised of well-characterized participants who undergo cognitive testing and physical exams every 2 years (Bettcher et al., 2018). The Wisconsin ADRC has a cohort of initially cognitively-unimpaired, asymptomatic middle-aged (between 45 and 65) adults with a similar study design to WRAP [the **I**nvestigating **M**emory in **P**reclinical **A**D-**C**auses and **T**reatments (IMPACT) cohort] (Racine et al., 2016; Darst et al., 2017; Vogt et al., 2018). From the IMPACT cohort, we identified 154 self-reported non-Hispanic white participants with cross-sectional CSF biomarker and metabolomic data available between 2010 to 2017. As with WRAP, the sample size for other racial/ethnic groups was too small (*n* < 10) to include in the analyses.

### Standard protocol approvals, registration, and patients

This study was conducted with the approval of the University of Wisconsin Institutional Review Board, and all participants provided signed informed consent before participation.

### CSF sample collection and biomarkers quantification

Fasting CSF samples were collected via lumbar puncture using a Sprotte 25- or 24-gauge spinal needle at the L3/4 or L4/5 interspace with gentle extraction into polypropylene syringes. More details can be found in the previous study (Darst et al., 2017). The CSF collection for WRAP and the Wisconsin ADRC followed the same protocol, and the lumbar puncture for both studies was performed by the same group of well-trained individuals.

All CSF samples were batched together and assayed for the NTK biomarkers at the Clinical Neurochemistry Laboratory, University of Gothenburg, using the same lot of reagents, under strict quality control procedures. The immunoassays of Elecsys^®^ Aβ(1–42), P-tau(181P), and T-tau, as well as S100b and IL-6, were performed on a cobas e 601 analyzer (Hulle et al., 2021). The remaining NTK panel was assayed on a cobas e 411 analyzer including Aβ(1–40), α-synuclein, GFAP, YKL-40, sTREM2, NfL, and neurogranin (Hulle et al., 2021).

### CSF metabolomic profiling and quality control

Cerebrospinal fluid metabolomic analyses and quantification were performed in one batch by Metabolon (Durham, NC) using an untargeted approach, based on Ultrahigh Performance Liquid Chromatography-Tandem Mass Spectrometry platform (UPLC-MS/MS) (Evans et al., 2014). Details of the metabolomic profiling regarding sample preparation, metabolite extraction, derivatization, separation and detection, and raw data processing were described in an earlier study (Darst et al., 2019).

A total of 412 CSF metabolites were identified and quality control procedures were performed. First, 46 metabolites missing for at least 80% of the individuals were excluded. Then the values for each of the remaining metabolites were scaled so that the median equaled 1. Two metabolites with an interquartile range (IQR) of zero were excluded and no metabolites had zero variability between individuals. Log10 transformation was applied to normalize the data. After quality control, 269 metabolites with known biochemical names remained for this investigation. The missing percentage of each metabolite in WRAP and Wisconsin ADRC is available in Supplementary Table 1.

### Genotyping and quality control

In the WRAP participants, DNA was extracted from whole blood using the PUREGENE^®^ DNA Isolation Kit, and the concentrations were quantified using the Invitrogen™ Quant-iT™ PicoGreen™ dsDNA Assay Kit. More details can be found in the previous study (Darst et al., 2019). Genotyping data were generated by the University of Wisconsin Biotechnology Center using the Illumina Multi-Ethnic Genotyping Array for 1,340 individuals originally. Quality control procedures have been described previously (Darst et al., 2019). Briefly, samples and variants with missingness >5% and samples with inconsistent genetic and self-reported sex were removed. The resulting 1,198 samples from European ancestry individuals and 898,220 variants were then imputed using the Michigan Imputation Server and the HRC reference panel. Variants with a low imputation quality score (*R*2 < 0.8), with a low minor allele frequency (MAF) (<0.001), or out of HWE were removed. The genetic ancestry was assessed by using Principal Components Analysis in Related Samples (PC-AiR) because of the sibling relationships present in the WRAP cohort.

Genetic data in the Wisconsin ADRC were generated from DNA extracted from blood samples at baseline and genotyped with either the Infinium OmniExpressExome-8 Kit or the Infinium Global Screening Array-24 Kit. Genetic data for the Wisconsin ADRC underwent the same quality control (QC) and imputation as the WRAP data except samples and SNPs missing in >2% were excluded and HWE threshold was *p* < 1e-6 due to differences in sample sizes and the number of SNPs between the two cohorts.

### Statistical analysis

#### Metabolome-wide association study

A metabolome-wide association study (MWAS) was conducted in the WRAP cohort between 269 individual CSF metabolites and 13 CSF NTK biomarkers using linear mixed-effects regression models with random intercepts to account for repeated measures and family relationships (10 families with two or more siblings) and fixed effects for age at CSF collection, sex, and years of education. Replication of each CSF metabolite significantly associated with one or more biomarkers in WRAP was then conducted in the Wisconsin ADRC cohort using linear regression adjusting for the same covariates. Both Bonferroni and false discovery rate (FDR) methods were used to correct the *p*-values for multiple testing; the FDR corrected q value was used to determine statistical significance in each analysis. Potential functional pathways of the replicated significant metabolites were identified by pathway analyses using the web-based software Metabo-analyst 5.0 (Pang et al., 2021) based on the Kyoto Encyclopedia of Genes and Genomes (KEGG) Homo sapiens pathway. The hypergeometric test and relative-betweenness centrality were employed to evaluate the pathway importance, and the pathways with an impact score ≥0.1 were selected.

#### Prediction performance and elastic net regression

The variance for each biomarker explained by its corresponding significant metabolites was evaluated using r^2^ in the combined and imputed cohorts of WRAP and the Wisconsin ADRC. For this analysis, we only included the first available measures of independent participants from WRAP. Since the number of significant metabolites for each biomarker was large and some of the metabolites were highly correlated, elastic net regression (Zou and Hastie, 2005) was employed to select the important independent metabolites. Then the r^2^ of elastic net-selected metabolites was re-calculated. For each biomarker, we fit three types of models, the (1) base model, which only included the demographics of age, sex, years of education, and indicator of cohort, (2) metabolite model, which included the demographics in the base model plus all the replicated significant metabolites, and (3) elastic net-selected metabolite model, which contained the demographics and elastic-net-selected metabolites.

### Mendelian randomization

In our analysis, we employed Mendelian randomization (MR) (Sanderson et al., 2022) which uses genetic variation as an instrumental variable to assess the causal relationship about whether metabolites influence the CSF NTK biomarkers. The genome-wide significant SNPs (*p* < 5 × 10^–8^) from a previous genome-wide meta-analysis of CSF metabolites (Panyard et al., 2021) were extracted for each elastic net-selected metabolite (5,863 SNPs for 52 metabolites). These SNPs (or the top 100 SNPs if there were more than 100 genome-wide significant SNPs for a metabolite) were used as instrumental variables (IV) for the metabolite. Then we conducted one-sample MR analysis for each elastic net-selected metabolite-NTK biomarker association pair in the combined WRAP and Wisconsin ADRC cohort by using the summary statistics and individual-level genetic data of extracted SNPs. For each MR test, we first checked the strength of the IVs using F statistics. Typically, an IV with an F statistic greater than 10 is considered to be strong, while instruments with F statistics below 10 are considered to be weak (Stock et al., 2002). Next, the estimated (or less confounded) beta and *p*-values for the effect of the metabolite on the NTK biomarker were calculated using the two-stage least squares method if the IVs were strong and correlated, but using the limited information maximum likelihood (LIML) for correlated IVs that were relatively weak (Chao and Swanson, 2005; Wooldridge, 2010). The confidence intervals (CI) of the point estimates from both LIML and another conditional likelihood ratio (CLR) method, which is robust to weak IVs (Moreira, 2003), were compared and only significant results with CIs in the same direction and with a similar range of effect size between these two methods were considered as evidence of a causal effect. The Bonferroni corrected *p*-value < 0.05 based on the number of all MR tests performed was used to determine significance. The MR analysis was conducted using the R package “ivmodel” (Kang et al., 2021).

### Data availability

The genetic data have been uploaded to the National Institute on Aging Genetics of Alzheimer’s Disease Data Storage Site (NIAGADS) and metabolomic data are being uploaded to Accelerating Medicines Partnership Program for Alzheimer’s Disease (AMP-AD).

## Results

### Participant characteristics

Characteristics of the WRAP and Wisconsin ADRC participants can be found in Table 1. In the WRAP cohort, just over one third of the participants had one CSF sample, almost one third had two CSF samples collected approximately 2 years apart, and just over one third had three CSF samples collected approximately every 2 years. The first available measures were used to calculate the summary participant characteristics. In the Wisconsin ADRC cohort, only one sample was available for each participant. In both studies, the CSF was collected between 2010 and 2017. Among 161 WRAP participants, the mean age and education level were 62.1 and 16.2 years, respectively. The mean age and years of education in the Wisconsin ADRC were 58.1 and 16.2, respectively. Females comprised 65.2% of WRAP participants and 68.8% of the Wisconsin ADRC. In the longitudinal WRAP cohort, 80.1, 16.8, and 3.1% of participants were cognitively unimpaired stable, unimpaired declining, or had clinical MCI, respectively. Four participants progressed from cognitively unimpaired stable to unimpaired declining by the last CSF collection 2–4 years later. All participants in the Wisconsin ADRC were cognitively unimpaired stable. The mean values of each biomarker are also listed in Table 1.

**TABLE 1:** Sample characteristics of WRAP and Wisconsin ADRC participants.

	WRAP* *N* = 161		Wisconsin ADRC *N* = 154	
	Mean	SD	Mean	SD
Age	62.1	6.5	58.1	5.5
Years of education	16.2	2.2	16.2	2.3
P-tau	17.5	6.4	15.9	5.8
T-tau	200.6	67.3	184.5	69.3
Aβ42	895.4	369.4	942.6	363.1
Aβ40	14336.1	4497.9	13897.4	4742.3
NfL	87.2	38.0	83.3	80.7
Neurogranin	798.6	307.5	728.2	286.7
YKL-40	144.6	48.3	128.7	39.1
S100b	1.1	0.3	1.2	0.3
GFAP	8.6	2.9	8.6	3.3
sTREM2	7.9	2.4	7.6	2.1
IL-6	4.3	2.6	5.2	3.7
α-synuclein	157.4	65.1	146.2	64.1
	***N***	**%**	***N***	**%**
**Sex**				
Female	105	65.2	106	68.8
Male	56	34.8	48	31.2
**Cognitive status**				
Unimpaired stable	129	80.1	154	100
Unimpaired declining	27	16.8		
Clinical MCI	5	3.1		
**No. of samples per individual**				
1	56	34.8	154	100
2	45	27.9		
3	55	34.2		
4	5	3.1		

*The summary statistics of WRAP were based on each individual’s first available CSF collection where both metabolomics and biomarkers were measured.

### MWAS detects significant associations between CSF metabolites and biomarkers of AD pathology

**FIGURE 1:**
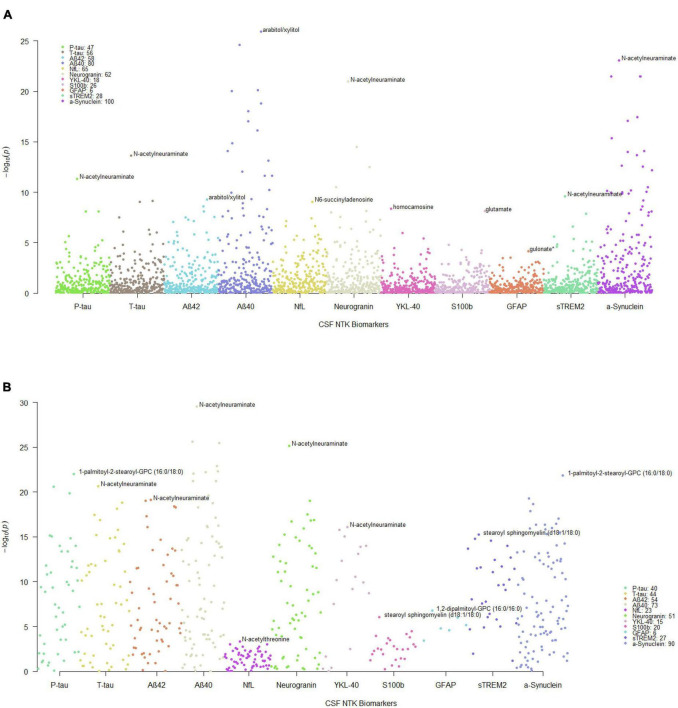
Manhattan plots of MWAS results between CSF metabolites and CSF NTK biomarkers. Each dot represents a metabolite and the different colors represent the CSF NTK biomarkers (x-axis) in **(A)** WRAP and **(B)** the Wisconsin ADRC (only significant metabolites after FDR correction in WRAP were included). The –log_10_(*p*-value) is shown on the y-axis. The legend box indicates the number of metabolites that were significant after FDR correction for each NTK biomarker. The annotated metabolites in the figures represent the most significant metabolite associated with each NTK biomarker.

**FIGURE 2:**
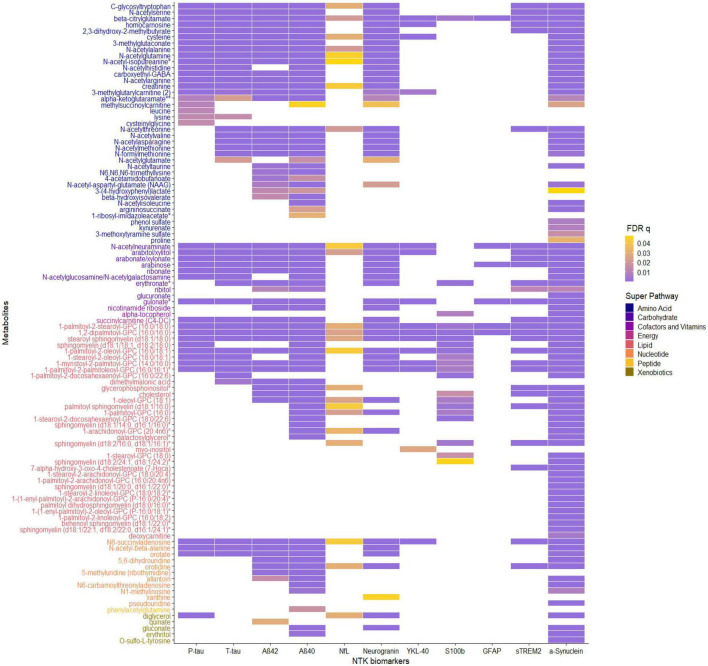
The association patterns between significant CSF metabolites and CSF NTK biomarkers in Wisconsin-ADRC. Each cell represents the association of a CSF metabolite with a biomarker. The color scale indicates the magnitude of the FDR *q*-values. The metabolites are also grouped and colored based on their super pathway.

The significant MWAS results in WRAP and the Wisconsin ADRC are summarized in Figure 1. In WRAP, a large number of CSF metabolites reached the significance threshold after FDR correction (Figure 1A). 47 metabolites were associated with P-tau, 56 were associated with T-tau, 58 were associated with Aβ42, 80 were associated with Aβ40, 65 were associated with NfL, and 62 were associated with neurogranin. However, no metabolites were associated with the ratio of Aβ42/40 or IL-6. Many of the metabolites that were significant in WRAP were also significant in the Wisconsin ADRC (Figure 1B). For example, among 47 significant metabolites for P-tau in WRAP, 40 metabolites were also significant in the Wisconsin ADRC. Table 2 shows the replication results for the top 10 significant CSF metabolite-biomarker associations (if there were 10 or more significant metabolites) in the Wisconsin ADRC. For example, the top three metabolites associated with P-tau and T-tau were 1-palmitoyl-2-stearoyl-GPC (16:0/18:0), N-acetylneuraminate, and C-glycosyltryptophan. N-acetylneuraminate and 1,2-dipalmitoyl-GPC (16:0/16:0) were the top two metabolites associated with Aβ42 and Aβ40. The top three metabolites associated with NfL were N-acetylthreonine, N-acetylalanine, and beta-citrylglutamate. N-acetylneuraminate, C-glycosyltryptophan, and N6-succinyladenosine were the top three metabolites for neurogranin. N-acetylneuraminate, 1,2-dipalmitoyl-GPC (16:0/16:0), and stearoyl sphingomyelin (d18:1/18:0) were the top three metabolites for YKL40. Stearoyl sphingomyelin (d18:1/18:0), 1-stearoyl-2-docosahexaenoyl-GPC (18:0/22:6), and 1-palmitoyl-2-oleoyl-GPC (16:0/18:1) were the top three metabolites associated with S100b. Only six metabolites were associated with GFAP, and the top three were 1,2-dipalmitoyl-GPC (16:0/16:0), 1-palmitoyl-2-stearoyl-GPC (16:0/18:0), and beta-citrylglutamate. For sTREM2, the top metabolites were stearoyl sphingomyelin (d18:1/18:0), 1,2-dipalmitoyl-GPC (16:0/16:0), and palmitoyl sphingomyelin (d18:1/16:0). Finally, for α-synuclein, the top three metabolites were 1-palmitoyl-2-stearoyl-GPC (16:0/18:0), 1,2-dipalmitoyl-GPC (16:0/16:0), and N-acetylneuraminate. The full results of WRAP and the Wisconsin ADRC can be found in Supplementary Tables 2–25. The association patterns between significant CSF metabolites and NTK biomarkers are provided in the Figure 2. The summary of the number of significant associations and the name of NTK biomarkers that were replicated in the Wisconsin ADRC are presented in Supplementary Table 26. Most of the significant metabolites were lipids, amino acids, and carbohydrates. For example, the lipid, 1,2-dipalmitoyl-GPC (16:0/16:0), the amino acid, beta-citrylglutamate, and the carbohydrate N-acetylneuraminate were strongly associated with almost every CSF NTK biomarker of AD. On the contrary, amino acids like kynurenate and proline were only significantly associated with α-synuclein.

**TABLE 2:** Top 10 significant CSF metabolites associated with each NTK biomarker in WRAP and replicated in the Wisconsin ADRC.

NTK Biomarker	Biochemical names	Compound ID	Beta	P	Adjusted P	FDR q	Super pathway	Sub-pathway
P-tau	1-palmitoyl-2-stearoyl-GPC (16:0/18:0)	52616	24.10	9.89E-23	4.65E-21	4.65E-21	Lipid	Phosphatidylcholine (PC)
	N-acetylneuraminate	32377	29.31	2.66E-21	1.25E-19	6.25E-20	Carbohydrate	Aminosugar metabolism
	C-glycosyltryptophan	48782	29.26	1.41E-20	6.62E-19	2.21E-19	Amino acid	Tryptophan metabolism
	1,2-dipalmitoyl-GPC (16:0/16:0)	19130	27.21	6.95E-16	3.27E-14	8.17E-15	Lipid	Phosphatidylcholine (PC)
	Stearoyl sphingomyelin (d18:1/18:0)	19503	26.20	9.38E-16	4.41E-14	8.81E-15	Lipid	Sphingolipid metabolism
	Arabitol/xylitol	48885	28.10	1.50E-15	7.04E-14	1.17E-14	Carbohydrate	Pentose metabolism
	Beta-citrylglutamate	54923	23.47	9.84E-15	4.62E-13	5.82E-14	Amino acid	Glutamate metabolism
	N-acetylserine	37076	35.24	9.90E-15	4.65E-13	5.82E-14	Amino acid	Glycine, serine and threonine metabolism
	Sphingomyelin (d18:1/18:1, d18:2/18:0)	37529	24.14	4.10E-14	1.93E-12	2.09E-13	Lipid	Sphingolipid metabolism
	N6-succinyladenosine	48130	22.14	4.44E-14	2.09E-12	2.09E-13	Nucleotide	Purine metabolism, adenine containing
T-tau	N-acetylneuraminate	32377	349.51	2.25E-21	1.26E-19	1.26E-19	Carbohydrate	Aminosugar metabolism
	1-palmitoyl-2-stearoyl-GPC (16:0/18:0)	52616	296.17	1.53E-19	8.54E-18	4.27E-18	Lipid	Phosphatidylcholine (PC)
	C-glycosyltryptophan	48782	336.75	7.77E-19	4.35E-17	1.45E-17	Amino acid	Tryptophan metabolism
	1,2-dipalmitoyl-GPC (16:0/16:0)	19130	343.88	3.72E-18	2.09E-16	5.21E-17	Lipid	Phosphatidylcholine (PC)
	N-acetylthreonine	33939	372.61	1.37E-17	7.68E-16	1.54E-16	Amino acid	Glycine, serine and threonine metabolism
	Arabitol/xylitol	48885	340.87	1.61E-16	9.00E-15	1.50E-15	Carbohydrate	Pentose metabolism
	Stearoyl sphingomyelin (d18:1/18:0)	19503	314.01	6.07E-16	3.40E-14	4.85E-15	Lipid	Sphingolipid metabolism
	N6-succinyladenosine	48130	275.91	1.19E-15	6.64E-14	8.30E-15	Nucleotide	Purine metabolism, adenine containing
	Erythronate*	42420	608.03	1.48E-15	8.30E-14	9.23E-15	Carbohydrate	Aminosugar metabolism
	Beta-citrylglutamate	54923	281.29	6.24E-15	3.49E-13	3.49E-14	Amino acid	Glutamate metabolism
Aβ42	N-acetylneuraminate	32377	1831.96	7.62E-20	4.42E-18	2.65E-18	Carbohydrate	Aminosugar metabolism
	1,2-dipalmitoyl-GPC (16:0/16:0)	19130	1922.65	9.15E-20	5.31E-18	2.65E-18	Lipid	Phosphatidylcholine (PC)
	1-palmitoyl-2-oleoyl-GPC (16:0/18:1)	52461	2019.63	4.04E-19	2.34E-17	7.50E-18	Lipid	Phosphatidylcholine (PC)
	1-palmitoyl-2-stearoyl-GPC (16:0/18:0)	52616	1608.22	5.17E-19	3.00E-17	7.50E-18	Lipid	Phosphatidylcholine (PC)
	1-myristoyl-2-palmitoyl-GPC (14:0/16:0)	19258	1779.94	5.29E-18	3.07E-16	6.13E-17	Lipid	Phosphatidylcholine (PC)
	Stearoyl sphingomyelin (d18:1/18:0)	19503	1735.41	7.98E-17	4.63E-15	7.72E-16	Lipid	Sphingolipid metabolism
	N-acetylserine	37076	2220.93	1.96E-15	1.14E-13	1.63E-14	Amino acid	Glycine, serine and threonine metabolism
	Arabitol/xylitol	48885	1732.68	2.00E-14	1.16E-12	1.45E-13	Carbohydrate	Pentose metabolism
	N-acetylthreonine	33939	1832.87	3.07E-14	1.78E-12	1.98E-13	Amino acid	Glycine, serine and threonine metabolism
	1-palmitoyl-2-palmitoleoyl-GPC (16:0/16:1)*	52470	1793.19	3.46E-14	2.01E-12	2.01E-13	Lipid	Phosphatidylcholine (PC)
Aβ40	N-acetylneuraminate	32377	27190.93	2.92E-30	2.34E-28	2.34E-28	Carbohydrate	Aminosugar metabolism
	1,2-dipalmitoyl-GPC (16:0/16:0)	19130	27204.24	2.46E-26	1.96E-24	9.51E-25	Lipid	Phosphatidylcholine (PC)
	1-palmitoyl-2-stearoyl-GPC (16:0/18:0)	52616	23086.44	3.57E-26	2.85E-24	9.51E-25	Lipid	Phosphatidylcholine (PC)
	1-stearoyl-2-oleoyl-GPC (18:0/18:1)	52438	27203.88	1.22E-23	9.72E-22	2.43E-22	Lipid	Phosphatidylcholine (PC)
	1-palmitoyl-2-oleoyl-GPC (16:0/18:1)	52461	27594.67	4.59E-23	3.67E-21	7.35E-22	Lipid	Phosphatidylcholine (PC)
	N-acetylserine	37076	34639.61	5.99E-23	4.79E-21	7.99E-22	Amino acid	Glycine, serine and threonine metabolism
	Stearoyl sphingomyelin (d18:1/18:0)	19503	24933.03	8.41E-23	6.73E-21	9.61E-22	Lipid	Sphingolipid metabolism
	Arabitol/xylitol	48885	26504.66	6.06E-22	4.85E-20	5.64E-21	Carbohydrate	Pentose metabolism
	1-myristoyl-2-palmitoyl-GPC (14:0/16:0)	19258	24513.57	6.34E-22	5.07E-20	5.64E-21	Lipid	Phosphatidylcholine (PC)
	Erythronate*	42420	47037.89	2.43E-20	1.94E-18	1.94E-19	Carbohydrate	Aminosugar metabolism
NfL	N-acetylthreonine	33939	194.81	4.51E-04	2.93E-02	2.09E-02	Amino acid	Glycine, serine and threonine metabolism
	N-acetylalanine	1585	262.23	9.53E-04	6.19E-02	2.09E-02	Amino acid	Alanine and aspartate metabolism
	Beta-citrylglutamate	54923	150.23	9.66E-04	6.28E-02	2.09E-02	Amino acid	Glutamate metabolism
	Arabitol/xylitol	48885	165.78	1.70E-03	1.10E-01	2.26E-02	Carbohydrate	Pentose metabolism
	1-palmitoyl-GPC (16:0)	33955	142.17	1.74E-03	1.13E-01	2.26E-02	Lipid	Lysophospholipid
	1,2-dipalmitoyl-GPC (16:0/16:0)	19130	154.06	2.68E-03	1.74E-01	2.53E-02	Lipid	Phosphatidylcholine (PC)
	1-oleoyl-GPC (18:1)	48258	136.99	2.72E-03	1.77E-01	2.53E-02	Lipid	Lysophospholipid
	Stearoyl sphingomyelin (d18:1/18:0)	19503	145.43	3.35E-03	2.18E-01	2.72E-02	Lipid	Sphingolipid metabolism
	Orotidine	35172	129.73	4.56E-03	2.96E-01	3.00E-02	Nucleotide	Pyrimidine metabolism, orotate containing
	Cysteine	1868	187.12	4.98E-03	3.24E-01	3.00E-02	Amino acid	Methionine, cysteine, SAM and Taurine metabolism
Neurogranin	N-acetylneuraminate	32377	1574.23	6.97E-26	4.32E-24	4.32E-24	Carbohydrate	Aminosugar metabolism
	C-glycosyltryptophan	48782	1442.13	9.29E-20	5.76E-18	2.88E-18	Amino acid	Tryptophan metabolism
	N6-succinyladenosine	48130	1234.78	3.19E-18	1.98E-16	6.60E-17	Nucleotide	Purine metabolism, adenine containing
	1-palmitoyl-2-stearoyl-GPC (16:0/18:0)	52616	1212.87	1.38E-17	8.53E-16	1.89E-16	Lipid	Phosphatidylcholine (PC)
	Arabitol/xylitol	48885	1470.51	1.53E-17	9.48E-16	1.89E-16	Carbohydrate	Pentose metabolism
	N-acetylthreonine	33939	1549.35	1.83E-17	1.13E-15	1.89E-16	Amino acid	Glycine, serine and threonine metabolism
	Erythronate*	42420	2626.14	1.19E-16	7.40E-15	1.06E-15	Carbohydrate	Aminosugar metabolism
	1,2-dipalmitoyl-GPC (16:0/16:0)	19130	1370.31	5.60E-16	3.47E-14	4.34E-15	Lipid	Phosphatidylcholine (PC)
	1-palmitoyl-GPC (16:0)	33955	1202.61	1.09E-15	6.78E-14	7.54E-15	Lipid	Lysophospholipid
	N-acetylserine	37076	1719.96	2.67E-15	1.65E-13	1.65E-14	Amino acid	Glycine, serine and threonine metabolism
YKL-40	N-acetylneuraminate	32377	162.38	7.62E-17	1.37E-15	1.37E-15	Carbohydrate	Aminosugar metabolism
	1,2-dipalmitoyl-GPC (16:0/16:0)	19130	169.92	1.71E-16	3.08E-15	1.54E-15	Lipid	Phosphatidylcholine (PC)
	Stearoyl sphingomyelin (d18:1/18:0)	19503	160.90	8.86E-16	1.60E-14	5.32E-15	Lipid	Sphingolipid metabolism
	1-palmitoyl-2-stearoyl-GPC (16:0/18:0)	52616	140.55	1.05E-14	1.89E-13	4.65E-14	Lipid	Phosphatidylcholine (PC)
	1-palmitoyl-2-oleoyl-GPC (16:0/18:1)	52461	171.39	1.29E-14	2.32E-13	4.65E-14	Lipid	Phosphatidylcholine (PC)
	Arabitol/xylitol	48885	162.00	7.35E-14	1.32E-12	2.21E-13	Carbohydrate	Pentose metabolism
	1-myristoyl-2-palmitoyl-GPC (14:0/16:0)	19258	147.07	4.57E-13	8.22E-12	1.17E-12	Lipid	Phosphatidylcholine (PC)
	N6-succinyladenosine	48130	121.32	2.81E-11	5.06E-10	6.32E-11	Nucleotide	Purine metabolism, adenine containing
	Cysteine	1868	181.51	6.78E-11	1.22E-09	1.36E-10	Amino acid	Methionine, cysteine, SAM and Taurine metabolism
	1-palmitoyl-2-palmitoleoyl-GPC (16:0/16:1)*	52470	148.93	1.16E-10	2.09E-09	2.09E-10	Lipid	Phosphatidylcholine (PC)
S100b	Stearoyl sphingomyelin (d18:1/18:0)	19503	0.81	8.99E-07	2.34E-05	2.34E-05	Lipid	Sphingolipid metabolism
	1-stearoyl-2-docosahexaenoyl-GPC (18:0/22:6)	52611	0.54	3.45E-05	8.97E-04	4.49E-04	Lipid	Phosphatidylcholine (PC)
	1-palmitoyl-2-oleoyl-GPC (16:0/18:1)	52461	0.73	6.95E-05	1.81E-03	6.03E-04	Lipid	Phosphatidylcholine (PC)
	1,2-dipalmitoyl-GPC (16:0/16:0)	19130	0.67	1.07E-04	2.79E-03	6.96E-04	Lipid	Phosphatidylcholine (PC)
	1-palmitoyl-2-docosahexaenoyl-GPC (16:0/22:6)	52610	0.50	1.59E-04	4.14E-03	8.27E-04	Lipid	Phosphatidylcholine (PC)
	Sphingomyelin (d18:1/18:1, d18:2/18:0)	37529	0.60	2.07E-04	5.39E-03	8.99E-04	Lipid	Sphingolipid metabolism
	Erythronate*	42420	1.18	3.28E-04	8.52E-03	1.22E-03	Carbohydrate	Aminosugar metabolism
	Palmitoyl sphingomyelin (d18:1/16:0)	37506	0.55	7.47E-04	1.94E-02	2.43E-03	Lipid	Sphingolipid metabolism
	1-palmitoyl-2-stearoyl-GPC (16:0/18:0)	52616	0.50	9.45E-04	2.46E-02	2.73E-03	Lipid	Phosphatidylcholine (PC)
GFAP	Sphingomyelin (d18:2/16:0, d18:1/16:1)*	42459	0.44	1.53E-03	3.97E-02	3.96E-03	Lipid	Sphingolipid metabolism
	1,2-dipalmitoyl-GPC (16:0/16:0)	19130	10.08	1.76E-07	1.06E-06	1.06E-06	Lipid	Phosphatidylcholine (PC)
	1-palmitoyl-2-stearoyl-GPC (16:0/18:0)	52616	8.07	9.91E-07	5.95E-06	2.97E-06	Lipid	Phosphatidylcholine (PC)
	Beta-citrylglutamate	54923	7.74	6.92E-06	4.15E-05	1.38E-05	Amino acid	Glutamate metabolism
	N-acetylneuraminate	32377	8.00	1.59E-05	9.56E-05	2.39E-05	Carbohydrate	Aminosugar metabolism
	Gulonate*	46957	6.71	2.75E-05	1.65E-04	3.30E-05	Cofactors and vitamins	Ascorbate and aldarate metabolism
	Arabinose	575	9.80	3.71E-04	2.22E-03	3.71E-04	Carbohydrate	Pentose metabolism
sTREM2	Stearoyl sphingomyelin (d18:1/18:0)	19503	9.59	5.72E-16	1.60E-14	1.60E-14	Lipid	Sphingolipid metabolism
	1,2-dipalmitoyl-GPC (16:0/16:0)	19130	9.81	1.59E-15	4.45E-14	2.23E-14	Lipid	Phosphatidylcholine (PC)
	Palmitoyl sphingomyelin (d18:1/16:0)	37506	9.18	2.52E-15	7.05E-14	2.35E-14	Lipid	Sphingolipid metabolism
	1-palmitoyl-2-oleoyl-GPC (16:0/18:1)	52461	10.20	9.68E-15	2.71E-13	6.78E-14	Lipid	Phosphatidylcholine (PC)
	Cholesterol	63	9.08	2.08E-14	5.83E-13	1.17E-13	Lipid	Sterol
	1-palmitoyl-2-stearoyl-GPC (16:0/18:0)	52616	7.89	2.11E-13	5.90E-12	9.83E-13	Lipid	Phosphatidylcholine (PC)
	sphingomyelin (d18:2/16:0, d18:1/16:1)*	42459	7.19	3.53E-13	9.88E-12	1.41E-12	Lipid	Sphingolipid metabolism
	C-glycosyltryptophan	48782	8.51	2.09E-12	5.86E-11	7.33E-12	Amino acid	Tryptophan metabolism
	N-acetylneuraminate	32377	8.35	2.55E-12	7.13E-11	7.92E-12	Carbohydrate	Aminosugar metabolism
	1-myristoyl-2-palmitoyl-GPC (14:0/16:0)	19258	8.45	3.18E-12	8.90E-11	8.90E-12	Lipid	Phosphatidylcholine (PC)
**α -synuclein**	1-palmitoyl-2-stearoyl-GPC (16:0/18:0)	52616	306.42	1.42E-22	1.42E-20	1.42E-20	Lipid	Phosphatidylcholine (PC)
	1,2-dipalmitoyl-GPC (16:0/16:0)	19130	337.75	5.02E-20	5.02E-18	2.51E-18	Lipid	Phosphatidylcholine (PC)
	N-acetylneuraminate	32377	315.50	2.29E-19	2.29E-17	7.64E-18	Carbohydrate	Aminosugar metabolism
	Stearoyl sphingomyelin (d18:1/18:0)	19503	316.41	1.31E-18	1.31E-16	3.28E-17	Lipid	Sphingolipid metabolism
	1-stearoyl-2-oleoyl-GPC (18:0/18:1)	52438	334.85	8.69E-18	8.69E-16	1.74E-16	Lipid	Phosphatidylcholine (PC)
	C-glycosyltryptophan	48782	304.78	3.58E-17	3.58E-15	5.82E-16	Amino acid	Tryptophan metabolism
	Palmitoyl sphingomyelin (d18:1/16:0)	37506	297.65	4.24E-17	4.24E-15	5.82E-16	Lipid	Sphingolipid metabolism
	N-acetylthreonine	33939	343.71	4.65E-17	4.65E-15	5.82E-16	Amino acid	Glycine, serine and threonine metabolism
	N6-succinyladenosine	48130	266.82	9.85E-17	9.85E-15	1.09E-15	Nucleotide	Purine metabolism, adenine containing
	1-myristoyl-2-palmitoyl-GPC (14:0/16:0)	19258	300.75	1.17E-16	1.17E-14	1.17E-15	Lipid	Phosphatidylcholine (PC)

*Indicates a compound that has not been confirmed based on a standard, but Metabolon was confident in its identity.

The functional pathways for replicated significant metabolites with known human metabolome database (HMDB) IDs for each CSF NTK biomarker are shown in Supplementary Table 27. Two significant metabolites, 1,2-dipalmitoyl-GPC (16:0/16:0) and 1-oleoyl-GPC (18:1), were enriched in the glycerophospholipid metabolism pathway for most biomarkers. Other pathways such as pyrimidine metabolism (including orotate and orotidine), ascorbate and aldarate metabolism (including gulonate and glucuronate), arginine biosynthesis (including N-acetylglutamate and argininosuccinate), and pentose and glucuronate interconversions (also including gulonate and glucuronate) may also be of interest.

### Prediction performance for CSF biomarkers of AD pathology improved after addition of CSF metabolites

The prediction performance of replicated significant metabolites was measured by r^2^ and presented in Table 3. The r^2^ of the base models, which only included the demographic variables, ranged from 0.01 to 0.25. Adding the replicated significant metabolites increased the r^2^ substantially for each biomarker, ranging from 0.23 to 0.74. The elastic net regression further prioritized candidate metabolites associated with each biomarker. For example, 14 of the original 40 significant metabolites were selected by the elastic net as important independent metabolites for P-tau. Initially, 40 significant metabolites explained about 72% of the variance in P-tau; the 22 elastic net-selected metabolites still explained 67% of the variance.

**TABLE 3:** Prediction performance (r^2^) of metabolites in the combined cohort of WRAP and Wisconsin ADRC.

NTK biomarkers	*N* *	Base model r2	Metabolite model r2	Number of input metabolites	Number of elastic net selected metabolites	Elastic net selected metabolites model r2
P-tau	295	0.11	0.71	40	14	0.67
T-tau	296	0.10	0.72	44	19	0.70
Aβ42	296	0.01	0.52	54	47	0.52
Aβ40	293	0.04	0.80	73	71	0.80
NfL	297	0.10	0.24	23	15	0.21
Neurogranin	297	0.07	0.77	51	32	0.76
YKL-40	297	0.25	0.56	15	10	0.56
S100	296	0.05	0.23	20	4	0.19
GFAP	297	0.17	0.35	6	5	0.35
sTREM2	297	0.05	0.53	27	14	0.52
α-synuclein	297	0.03	0.74	90	30	0.67

Variables included in the base model were age, sex, years of education and cohort.

Variables included in the metabolite model were age, sex, years of education, cohort and all replicated significant metabolites for each biomarker.

Variables included in the metabolite model were age, sex, years of education, cohort and elastic net-selected metabolites.

*The sample sizes were different because of the missingness of metabolites.

### Mendelian randomization detects metabolites with a potential causal effect on CSF biomarkers of AD pathology

According to the F statistics, we employed the LIML method for MR. The full results of the test statistics are provided in Supplementary Table 28. After checking for consistency of the CIs for the LIML and CLR methods, the significant and consistent MR results are displayed in Table 4, showing metabolites with a potential causal effect on the NTK biomarker based on instrumental variables formed by genome-wide significant SNPs. For example, we observed a positive causal association between palmitoyl sphingomyelin (d18:1/16:0) and sTREM2.

**TABLE 4:** Significant Mendelian randomization results after Bonferroni correction.

NTK biomarkers	Metabolite	Compound ID	nSNPs	nRegions (1Mbps)	F statistics	LIML estimate	LIML 95% CI		LIML *p*	Adjusted *p*
sTREM2	Palmitoyl sphingomyelin (d18:1/16:0)	37506	67	21	1.97	12.97	9.14	16.80	2.09E−10	2.50E−08
Aβ40	Erythritol	20699	100	34	6.09	12499.02	7539.22	17458.82	1.40E−06	1.61E−04
α-synuclein	Homocarnosine	1633	43	14	2.61	−123.04	−172.61	−73.47	1.96E−06	2.23E−04
T-tau	1-palmitoyl-2-stearoyl-GPC (16:0/18:0)	52616	19	8	2.81	319.63	187.99	451.28	3.21E−06	3.59E−04
α-synuclein	Erythritol	20699	100	34	5.95	172.93	99.70	246.16	5.69E−06	6.31E−04
Neurogranin	1-palmitoyl-2-stearoyl-GPC (16:0/18:0)	52616	19	8	2.82	1433.89	799.21	2068.57	1.37E−05	1.49E−03
Aβ40	1-myristoyl-2-palmitoyl-GPC (14:0/16:0)	19258	38	20	2.48	18666.39	10385.64	26947.14	1.43E−05	1.54E−03
T-tau	1-palmitoyl-2-stearoyl-GPC (16:0/18:0)	52616	19	8	2.79	28.06	15.50	40.62	1.70E−05	1.82E−03
α-synuclein	1-palmitoyl-2-stearoyl-GPC (16:0/18:0)	52616	19	8	2.82	268.34	137.78	398.90	7.16E−05	7.59E−03
Aβ40	1,2-dipalmitoyl-GPC (16:0/16:0)	19130	5	2	6.78	25973.09	12768.09	39178.09	1.41E−04	1.48E−02
Aβ40	Homocarnosine	1633	43	14	2.55	−6548.41	−9903.63	−3193.19	1.58E−04	1.65E−02
Neurogranin	Homocarnosine	1633	43	14	2.61	−444.47	−675.36	−213.58	1.93E−04	1.99E−02
Aβ40	Gulonate	46957	25	13	6.34	12658.31	6029.03	19287.58	2.17E−04	2.21E−02

The nSNPs refers to the number of SNPs in each IV, and the nRegions is the approximate number of regions defined by up to a 1Mbps of the SNPs. The F statistic represents the strength of the IV (strong IV F statistic > 10). The estimate of beta, confidence interval and *p*-values were all based on the limited information maximum likelihood (LIML) method.

## Discussion

In this analysis, we tested the associations between CSF metabolites and CSF NTK biomarkers representing different pathologies of AD in initially cognitively-unimpaired individuals. Significant metabolites were identified in the WRAP cohort using linear mixed effects regression and most of the metabolites were replicated in the Wisconsin ADRC cohort. The elastic net regression method reduced the number of CSF metabolites by selecting the important and independent metabolites for each CSF biomarker. This provides a smaller, more practical set of metabolites to focus on in future research. The results of the MR analyses suggested several metabolites that may play a causal role in AD pathology. A detailed look into these associations, such as the contributing genes and their corresponding functions, is worth exploring.

We have identified and replicated multiple CSF metabolites that were associated with CSF NTK biomarkers for AD pathology; most of these CSF metabolites were lipids, particularly sphingolipids, phosphatidylcholines, and lysophospholipids, which are all types of phospholipids. Phospholipids are a class of lipids that construct the cellular membranes and are involved in many complex activities of membrane proteins, receptors, enzymes, and ion channels in the cell or at the cell surface (Kosicek and Hecimovic, 2013). In the neurodegenerative brain, e.g., in the AD brain, which has suffered extensive damage, the compromise of the membrane functions is expected, explaining how phospholipids may be involved in AD pathology (Wong et al., 2017). Previous studies have demonstrated that various phospholipids such as phosphatidylcholines, sphingolipids, glycerophospholipids, and lysophospholipids have changed in the AD patient’s brain, CSF and blood when compared to healthy controls (Kosicek and Hecimovic, 2013; González-Domínguez et al., 2014; Kao et al., 2020). For example, a serum metabolomics study conducted by González-Domínguez et al. (2014) showed that the concentration of numerous phosphatidyl lipids, like 1,2-dipalmitoyl-GPC (16:0/16:0), 1-palmitoyl-2-linoleoyl-GPC (16:0/18:2), and 1-palmitoyl-2-oleoyl-GPC (16:0/18:1), and lysophosphatidylcholines, like 1-palmitoyl-GPC (16:0) and 1-stearoyl-GPC (18:0), were different in AD versus healthy controls. The 1,2-dipalmitoyl-GPC (16:0/16:0) phosphatidylcholine has also been suggested as one of three serum metabolites to predict AD development in MCI individuals (Orešič et al., 2011). Another brain metabolomics study found that higher levels of palmitoyl sphingomyelin (d18:1/16:0) and sphingomyelin (d18:1/18:1, d18:2/18:0) were associated with the severity of AD pathology at autopsy and AD progression across prodromal and preclinical stages (Varma et al., 2018). The stearoyl sphingomyelin (d18:1/18:0) was also significantly changed in the CSF with “AD-like pathology” that was dichotomized by Aβ42, T-tau, and P-tau levels (Koal et al., 2015). In summary, our results confirmed the importance of the previously identified lipids but also provided novel lipid findings for AD pathologies beyond the major established ones.

Another class of metabolites that are of potential interest are several carbohydrates like N-acetylneuraminate, arabitol/xylitol, arabinose, and erythronate. Among them, N-acetylneuraminate, also known as sialic acid, had a significant effect on most NTK biomarkers. In addition to our study, a previous study conducted by Nagata et al. (2018) in 2018 also showed that CSF N-acetylneuraminate was significantly increased in AD when compared to patients with idiopathic normal pressure hydrocephalus and was positively correlated with CSF P-tau (*r* = 0.55), as it was in our study. N-acetylneuraminate is an acetyl derivative of the amino sugar neuraminic acid, which occurs in many glycoproteins, glycolipids, and polysaccharides. Specifically, it is a functional and structural component of gangliosides, which are found predominantly in the nervous system and are abundant in the brain, especially in the grey matter (Palmano et al., 2015). Studies have shown that gangliosides play important roles in AD. For example, it has been suggested that GM1-ganglioside binds to Aß, and the resulted GAß has the capability to accelerate Aß assembly (Yanagisawa et al., 1995) and is the endogenous seed for amyloid fibral in the AD brain (Hayashi et al., 2004). The gangliosides also have important roles in organizing the lipid rafts, which integrate numerous types of lipid proteins involved in cell signaling, cell-cell adhesion, and intracellular vesicular trafficking (Nagata et al., 2018) and contain many AD-associated proteins such as amyloid precursor protein (APP) (Ehehalt et al., 2003). Furthermore, the gene *CD33*, which belongs to the sialic-acid-binding immunoglobulin-like lectin family, has been reported as a strong genetic locus associated with AD by GWASs (Bertram et al., 2008; Hollingworth et al., 2011; Naj et al., 2011) and has been suggested to impair the microglia-mediated Aβ clearance (Bradshaw et al., 2013; Griciuc et al., 2013; Jiang et al., 2014). Erythronate (erythronic acid) was previously identified as the main hallmark of pentose–phosphate pathway defects (Engelke et al., 2010), and consistent with abnormal function of pentose–phosphate pathway in certain regions of the AD-brain (Xu et al., 2016), and the upregulation of the pentose–phosphate pathway was reported in a previous study of mild cognitive impairment (MCI) participants that later progressed to AD (Orešič et al., 2011).

As mentioned above, a couple of metabolites were common to most of the AD pathologies defined by the CSF NTK biomarkers. On the contrary, some metabolites were unique to specific NTK biomarkers. For example, lipids like 1-palmitoyl-2-linoleoyl-GPC (16:0/18:2), 1-stearoyl-2-arachidonoyl-GPC (18:0/20:4), sphingomyelin (d18:1/20:0, d16:1/22:0) and sphingomyelin (d18:1/22:1, d18:2/22:0, d16:1/24:1) were only associated with α-synuclein. These metabolites may be helpful to study synaptic dysfunction and could potentially be used as biomarkers to differentiate AD pathologies.

The significant associations between a number of metabolites and both Aβ42 and Aβ40, but not with Aβ42/40 may indicate that the metabolites associated with Aβ42 and Aβ40 only influence the production of amyloid in general versus clearance of the pathological form, Aβ42. Our analysis also suggested that no metabolites were associated with IL-6, consistent with two other studies in the WRAP and Wisconsin ADRC cohorts that found no associations between twelve SM metabolites and IL-6 (Morrow et al., 2021), and no associations between a proteomic analysis of 915 proteins and IL-6 after multiple-testing correction (Johnson et al., 2020).

By utilizing Mendelian randomization, we found causal evidence for several of the associations between CSF metabolites and CSF NTK biomarkers. Among these metabolites, most of them were lipids, with some amino acids and cofactors/vitamins, and a xenobiotic metabolite, erythritol. Another metabolite of interest, homocarnosine, is an inhibitory neuromodulator synthesized in the neuron from gamma-aminobutyric acid (GABA) and histidine (Gujar et al., 2005). The level of human CSF homocarnosine declines drastically with age (Jansen et al., 2006) and was suggested to be related to AD through CSF protein glycation (Hipkiss, 2007). At the same time, GABA also plays an important role in the brain and may be related to AD (Govindpani et al., 2017).

This study has some limitations. First, the analysis only included non-Hispanic white individuals, so the results may not extrapolate to other racial/ethnic groups. Second, the sample sizes of both the WRAP and Wisconsin ADRC cohorts were relatively small and will need to be replicated in a larger independent sample. Although we excluded the metabolites with very high missingness (>80%), there were 10 metabolites with missing values in over 50% of individuals in WRAP and/or the Wisconsin ADRC (Supplementary Table 1). The missingness of these metabolites reduced the power and may have resulted in a failure to detect the association between them and biomarkers. The validation of these top hits using a targeted approach, more sophisticated statistical methods, and experiments *in vitro* are necessary for these results to be clinically relevant. Our analysis also excluded several metabolites with unknown biochemical names, but due to the development of Metabolon’s library and rapidly increasing studies in this area, it would be worthwhile to re-examine these metabolites when we have more knowledge. Finally, the MR conclusion can be nullified if the underlying assumptions are violated. For example, our conclusions may be sensitive to the presence of invalid IVs due to potential pleiotropy of metabolites. In general, the research confirmed that several novel metabolites changed along with AD CSF biomarkers and extended several developing and understudied AD pathologies, e.g., synaptic dysfunction, based on untargeted CSF metabolomics and will expand our knowledge of the biological mechanisms behind AD.

## Data availability statement

The genetic data presented in the study are deposited in the National Institute on Aging Genetics of Alzheimer’s Disease Data Storage Site (NIAGADS, https://www.niagads.org/home), accession number: NG00067. The metabolomic data presented in the study are deposited in Synapse (https://www.synapse.org/), project SynID: syn52222896.

## Ethics statement

The studies involving human participants were reviewed and approved by the University of Wisconsin Institutional Review Board. The patients/participants provided their written informed consent to participate in this study.

## Author contributions

RD conceived and designed the analysis, contributed data or analysis tools, performed the analysis, and wrote the manuscript. QL, HK, YD, and CV contributed data or analysis tools, modified the manuscript, and provided suggestion. IS, GK, NW, HZ, and KB collected the data, modified the manuscript, and provided suggestion. RA modified the manuscript and provided suggestion. CC, SA, and SJ collected the data. CE conceived and designed the analysis, collected the data, wrote the manuscript, and provided suggestion. All authors contributed to the article and approved the submitted version.
